# Mitochondrial transfer in cancer: mechanisms, immune evasion, and therapeutic opportunities

**DOI:** 10.1186/s44342-025-00064-1

**Published:** 2026-01-13

**Authors:** Hye In Ka, Hyun Goo Woo

**Affiliations:** 1 Department of Physiology, Ajou University School of Medicine, 164 Worldcup-Ro, Yeongtong-Gu, Suwon, Republic of Korea; 2 Department of Biomedical Science, Ajou University Graduate School, Suwon, Republic of Korea; 3 R&E Initiative for Advanced Precision Medicine, Ajou University Graduate School, BK21 Suwon, Republic of Korea

**Keywords:** Mitochondria, Mitochondrial Transfer, Cancer, Immune Evasion

## Abstract

Intercellular mitochondrial transfer (MT) is emerging as a transformative communication axis in cancer biology. Intact mitochondria or mitochondrial components can be exchanged between tumor cells, stromal elements, and immune cells via tunneling nanotubes, extracellular vesicles, cell fusion, or phagocytic uptake. This organelle exchange enables metabolic adaptation by restoring OXPHOS (oxidative phosphorylation), increasing ATP production, and enhancing survival in hostile environments. Conversely, tumor cells also hijack mitochondria from cytotoxic lymphocytes thereby undermining immune function and contributing to immune escape and tumor progression. These converging metabolic exchanges fuel immune evasion, metastatic potential, and resistance to chemotherapy, radiation, and immunotherapy. Cutting-edge tracing tools, including mitochondrial reporter proteins and single-cell mitochondrial genome lineage mapping, have uncovered MT events both in vitro and in vivo. Therapeutic strategies designed to block mitochondrial trafficking, inhibit nanotube formation or vesicle uptake, or enhance immune cell mitochondrial resilience hold promise for tumor sensitization and restoration of antitumor immunity. A deeper understanding of MT provides novel insight into cancer metabolism and intercellular communication, offering a foundation for future therapeutic innovation and potential clinical application as both a biomarker and a therapeutic target.

## Introduction

Mitochondria play a central role in regulating cellular energy metabolism, redox balance, and cell fate decisions between survival and death, yet their influence in cancer extends beyond the boundaries of a single cell [1, 2]. Evidence across model systems and human patient specimens shows that mitochondria can move between cells within the tumor microenvironment (TME) through tunneling nanotubes, extracellular vesicles, and fusion events. This trafficking, referred to as mitochondrial transfer (MT), restores respiration in metabolically compromised tumor cells, increases oxidative phosphorylation in nutrient-poor niches, and supports growth under genotoxic and metabolic stress. By providing intact respiratory capacity or key bioenergetic components, intercellular organelle exchange enables rapid metabolic reprogramming that favors survival, invasion, and treatment resistance [3].

The same process reshapes antitumor immunity. MT from lymphocytes to cancer cells can deplete immune cell reserves and weaken effector function, while the reverse flow of dysfunctional organelles from tumor to lymphocyte can elevate oxidative stress and blunt cytotoxicity. For instance, Ikeda et al. showed that tumor-derived mitochondria carrying mtDNA mutations are transferred to tumor-infiltrating lymphocytes (TILs), resulting in metabolic dysfunction, senescence, and poorer response to immune checkpoint inhibitors in melanoma and lung cancer patients [4]. Recent single-cell and tissue-level studies now connect these bidirectional exchanges to immune escape and therapeutic response, positioning MT as a communication axis that rewires the interface between cancer and the immune system [4, 5].

Methodological advances have made these intercellular relationships experimentally tractable in native contexts. Genetically encoded reporters and outer membrane tagging platforms allow visualization, immunocapture, and biochemical profiling of mitochondria transferred between defined cell populations directly from tissue. Endogenous mitochondrial DNA (mtDNA) variants function as natural barcodes that can be recovered in single cell assays to infer donor-recipient relationships without genetic engineering, and can be integrated with transcriptomic or chromatin accessibility readouts to map the state changes associated with transfer [6, 7]. Together, these tools provide engineering-free evidence for intercellular exchange, enable quantification at single-cell resolution, and open the door to causal experiments that link trafficking to function in primary human specimens.

These methodologies reveal translational opportunities while also beginning to elucidate how mitochondrial transfer shapes tumor metabolism and immunity. Strategies under investigation aim to interrupt physical conduits such as nanotubes, modulate upstream stress and cytoskeletal programs that drive intercellular connectivity, block vesicle uptake, or harden immune cell mitochondria against metabolic theft and oxidative injury [5, 8]. In parallel, measurements of mitochondrial chimerism and lineage relationships may serve as biomarkers of microenvironmental crosstalk and predictors of response to cytotoxic therapy, targeted agents, and immune checkpoint blockade. By viewing the tumor as a multicellular energetic network rather than a collection of isolated cells, interventions can be designed to starve malignant circuits of imported power while supporting the bioenergetic resilience of antitumor lymphocytes. This review synthesizes current mechanistic understanding of MT in cancer, highlights its role in immune evasion and therapy resistance, and outlines therapeutic avenues that leverage or disrupt this organelle-based communication to improve clinical outcomes [8].

This review summarizes the key mechanisms of MT in cancer development and progression, including tunneling nanotubes, extracellular vesicles, and cell fusion. We discuss their roles in metabolic reprogramming, immune modulation, and therapy resistance. Additionally, we explore current methods for tracing MT, therapeutic strategies aimed at disrupting these pathways, and the technical challenges ahead. A comprehensive understanding of MT at the mechanistic and translational levels may reveal novel diagnostic or therapeutic targets for cancer treatment.

### What’s new in this review

Unlike prior reviews that primarily summarized early discoveries of TNT-mediated mitochondrial trafficking, this work integrates recent mechanistic, immunological, and translational insights. We highlight new evidence identifying the bidirectional nature of MT between tumor and immune cells, its regulation by defined molecular pathways, and its implications for therapy resistance. Moreover, we introduce the recent methodological advances, including single-cell mtDNA lineage tracing and genetic reporters, which enable quantitative and causal investigation of MT in vivo. By bridging these mechanistic and technological advances with therapeutic strategies, this review provides a next-generation framework for understanding and targeting intercellular mitochondrial transfer in cancer.

## Mechanisms of MT in cancer

### Routes of intercellular MT

MT occurs through several pathways, with tunneling nanotubes (TNTs) among the most extensively studied. TNTs are thin, F-actin-rich cytoplasmic bridges that physically connect adjacent cells, allowing direct transport of intact mitochondria and mitochondrial DNA. These structures are often induced under stress conditions such as hypoxia or chemotherapy, facilitated by signaling molecules like Rho GTPases and TNFAIP2 [9, 10]. Stromal cells, such as cancer-associated fibroblasts and endothelial cells, frequently donate mitochondria to metabolically compromised tumor cells, enhancing mitochondrial respiration and therapy resistance [11]. Recent work has shown that malignant cells can hijack mitochondria from infiltrating T lymphocytes through TNT networks, depleting T cell mitochondrial reserves, thereby impairing effector function and facilitating immune evasion [4, 12, 13]. In hepatocellular carcinoma, hypoxic or nutrient-deprived stress upregulates HMGB1, RHOT1, and RAC1, promoting nanotube formation and enhancing MT between neighboring cells, restoring oxidative phosphorylation and enhancing survival, migration and invasion [14].

Extracellular vesicles (EVs), including exosomes and microvesicles, provide a contact-independent mechanism for mitochondrial transport by transporting mtDNA, mitochondrial proteins, and even whole mitochondria between cells [15–17]. For example, cancer-associated fibroblasts release EVs containing mtDNA, which are internalized by breast cancer cells, restoring respiration and promoting therapy resistance [18]. EV-mediated MT enables efficient delivery of metabolic "fuel" or signaling molecules across the TME without direct cell–cell contact. In addition to EVs, cell fusion represents a more infrequent but highly impactful mechanism, generating hybrid cells with shared cytoplasmic contents, including mitochondrial networks. Fusion between tumor cells and mesenchymal stem cells or macrophages can result in hybrids with enhanced oxidative phosphorylation and metastatic potential [19].

Furthermore, gap junctions, particularly involving connexin 43, facilitate close cell-to-cell adhesion and may enhance mitochondrial delivery through vesicular transfer rather than direct channel passage, facilitating vesicle-mediated delivery of mitochondria or mtDNA [20, 21]. Stressed cells can also release mitochondrial fragments into the extracellular space, which neighboring cells can internalize via endocytosis or phagocytosis. For instance, immune cells like macrophages may scavenge mitochondria from tumor cells, or conversely, tumor cells may acquire mitochondria from immune cells through macropinocytosis or other uptake mechanisms, as documented in leukemia models [22].

**Fig. 1:**
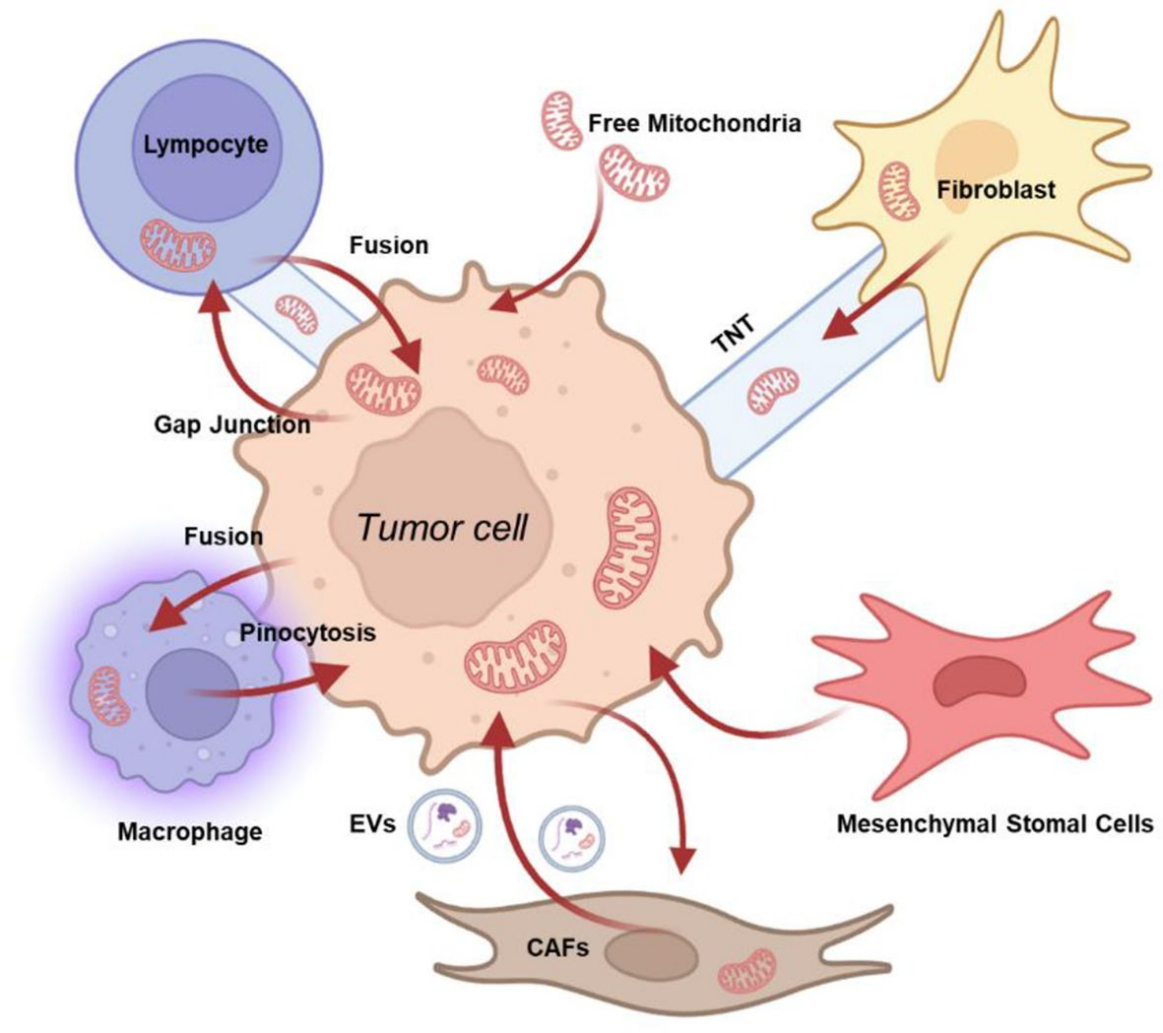
Graphical overview of the intercellular mitochondrial transfer between tumor and non-tumor cells. This schematic summarizes the major mechanisms of intercellular MT within the TME. Mitochondria can be exchanged bidirectionally between tumor cells and neighboring stromal or immune cells through multiple routes, including tunneling nanotubes (TNTs), extracellular vesicles (EVs), cell fusion, gap junctions, and phagocytic uptake. Fibroblasts, cancer-associated fibroblasts (CAFs), macrophages, lymphocytes, and mesenchymal stromal cells (MSCs) are depicted as representative partners engaged in MT, highlighting the complexity of intercellular organelle communication in the TME

These mechanisms are not mutually exclusive. A single tumor cell may exploit multiple MT routes in parallel, dynamically adapting to microenvironmental cues (Fig. 1). Collectively, this repertoire of mitochondrial trafficking strategies underscores the plasticity of cancer cells and their ability to reshape the tumor ecosystem to support survival, immune evasion, and cancer progression.

### Molecular regulation and cytoskeletal remodeling in MT

Although mitochondria can also be exchanged via extracellular vesicles and cell fusion, TNT-mediated transfer offers the most direct and experimentally tractable model to dissect the interplay between mitochondrial dynamics and cytoskeletal regulation. Therefore, this section highlights key molecular mediators that coordinate mitochondrial fission, trafficking, and TNT formation under oncogenic and stress conditions.

The regulation of mitochondrial transport via TNT is controlled by a complex network of proteins that manage the motility, division, and fusion of mitochondria, which ensures efficient transfer of functional mitochondria between cells [23]. This process is essential for cellular homeostasis and, in the case of cancer, intercellular mitochondrial transfer can provide metabolic advantages to tumor cells, promote survival under stress, and facilitate adaptation to surrounding TME [24]. Recent studies have highlighted the central role of mitochondrial proteins as adaptors linking mitochondria to cytoskeleton-related motor proteins, highlighting their roles in TNT structure and function [1, 24–26].

*MIRO1, also known as RHOT1,* is a mitochondrial Rho GTPase that acts as a calcium sensor, linking mitochondria to kinesin and dynein motor proteins [25, 27]. MIRO1 contains two GTPase domains and two EF-hand motifs that bind calcium, allowing the movement of mitochondria to be regulated according to changes in intracellular calcium levels. By interacting with motor proteins, MIRO1 enables both anterograde and retrograde mitochondrial movements within cells and via TNT along microtubules and actin filaments [26]. This bidirectional transport is essential not only for normal cellular function, but also for mitochondrial migration during stress or injury, which is evident in cancer and degenerative diseases [28]. In cancers including HCC, MIRO1 expression is frequently upregulated, enhancing mitochondrial transport and supporting tumor cell adaptation to metabolic stress and hypoxic conditions [24, 29]. Upregulation of MIRO1 is also associated with increased cell migration, invasion, and metastasis capacity and the ability of cancer cells to acquire mitochondria from surrounding substrates or immune cells [30]. Recent studies have shown that MIRO1 not only controls mitochondrial migration, but also functions in the formation and stabilization of TNT. It thus becomes a molecular bridge between mitochondria and cytoskeletons, and dysregulation can promote the migration and metastasis of cancer cells [31].

*Dynamin-related protein 1 (DRP1)* plays a crucial role in mitochondrial fission. It facilitates dividing mitochondria into smaller fragments that can be transported more easily through TNTs [32, 33]. DRP1 is recruited from the cytosol to the outer mitochondrial membrane to form oligomers and tighten the membrane to cause mitochondrial fission [34]. This process is tightly regulated by post-translational modifications, such as phosphorylation, SUMOylation, and ubiquitination, as well as by interactions with adaptor proteins like Fis1, MFF, MiD49, and MiD51 [35]. In cancers, DRP1 is frequently overexpressed and increases mitochondrial division, helping tumor cells to resist survival, growth, and apoptosis under metabolic stress [36, 37]. DRP1-induced mitochondrial division can also lead to the release of mitochondrial DNA and reactive oxygen species, which further affect TME and immune responses [38].

*Mitochondrial fission regulator 2 (MTFR2),* localized on the outer mitochondrial membrane, is another important protein that influences mitochondrial dynamics by promoting DRP1-dependent fission [39]. MTFR2 increases the number of mitochondria available for intercellular transfer, thereby meeting the high metabolic needs of rapidly growing tumor cells [40, 41]. MTFR2 interacts with DRP1 to prevent degradation in lysosomes and maintain mitochondrial division. Therefore, a recent study suggested that targeting with MTFR2 alone or with a DRP1 inhibitor (Mdivi-1) could be a novel approach to disrupting mitochondrial metastasis and metabolic adaptation in cancer [39]. The cooperative action of MIRO1, DRP1, and MTFR2 ensures efficient mitochondrial transfer and fission, allowing for the transfer of healthy mitochondria through TNTs [38, 42]. On the other hand, dysfunction of these regulatory proteins can inhibit mitochondrial movement and make cancer cells susceptible to metabolic stress and apoptosis [43].

The coordinated activity of MIRO1, DRP1, and MTFR2 is tightly regulated by upstream signaling pathways that respond to metabolic and inflammatory stress. Under hypoxic conditions, stabilization of HIF-1α enhances transcription of MIRO1 and DRP1, promoting mitochondrial fission and motility to facilitate TNT formation and organelle transfer. Simultaneously, cytokine-mediated activation of Rho-family GTPases, including RAC1 and CDC42, drives actin polymerization and filopodia extension, providing structural scaffolds for TNT elongation. TNFAIP2, a downstream effector of TNF-α/NF-κB and STAT3 signaling, also interacts with actin-binding proteins to stabilize these protrusions, linking inflammatory cues to cytoskeletal remodeling. Through these convergent pathways, external signals such as hypoxia and cytokine stimulation synchronize mitochondrial dynamics with the reorganization of the cytoskeleton, thereby promoting efficient intercellular mitochondrial transfer within the tumor microenvironment.

## Functional roles of MT in cancer

### Role in metabolic reprogramming

Beyond its structural and regulatory mechanisms, one of the most profound consequences of MT is the metabolic reprogramming of recipient cancer cells. Tumor cells in a nutrient-deprived or hypoxic microenvironment often suffer from mitochondrial dysfunction, resulting in insufficient oxidative phosphorylation and reduced ATP production. Acquisition of exogenous mitochondria can replenish functional electron transport chains and enzymes, thereby enhancing oxidative phosphorylation capacity and ATP production in recipient cells. This fuels tumor growth and improves survival under metabolic stress. When nutrient-deprived cells receive functional mitochondria or mtDNA from donor stromal or neural cells, they restore electron transport chain function and regain oxidative phosphorylation capacity, enabling survival and growth in harsh microenvironments [44]. In glioblastoma models, astrocytes transfer mitochondria to GBM cells in a contact-dependent manner. These recipient tumor cells exhibit increased proliferation and self-renewal in vitro and in orthotopic mouse models, and the acquisition correlates with accelerated tumor growth and shortened host survival [45]. This supports the notion that MT shifts metabolic balance in favor of oxidative fuel burning in otherwise glycolytic tumors.

Tissue-resident mesenchymal stromal cells (MSCs) or cancer-associated fibroblasts (CAF) also participate in MT. In ovarian cancer, carcinoma-associated mesenchymal stromal cells donate mitochondria preferentially to mitochondria-poor tumor cells thereby rescuing respiration and proliferation [46]. This mitochondrial donation increases heterogeneity within the tumor cell population, promoting metastatic spread and worsened survival in murine models. These results support the idea that MT acts as a metabolic rescue pathway that shifts cancer cell fueling from glycolysis toward oxidative phosphorylation, thereby enabling growth under stress. Paradoxically, the transfer can also occur in the opposite direction—from tumor to fibroblast—reprogramming stromal metabolism. This transfer reprograms fibroblasts metabolically, induces the expression of CAF markers, and stimulates the release of pro-tumorigenic cytokines, growth factors, and extracellular matrix components, converting normal fibroblasts into CAFs [47]. Beyond metabolic adaptation, MT also exerts profound immunological effects within the TME.

### Role in immune evasion

**Fig. 2:**
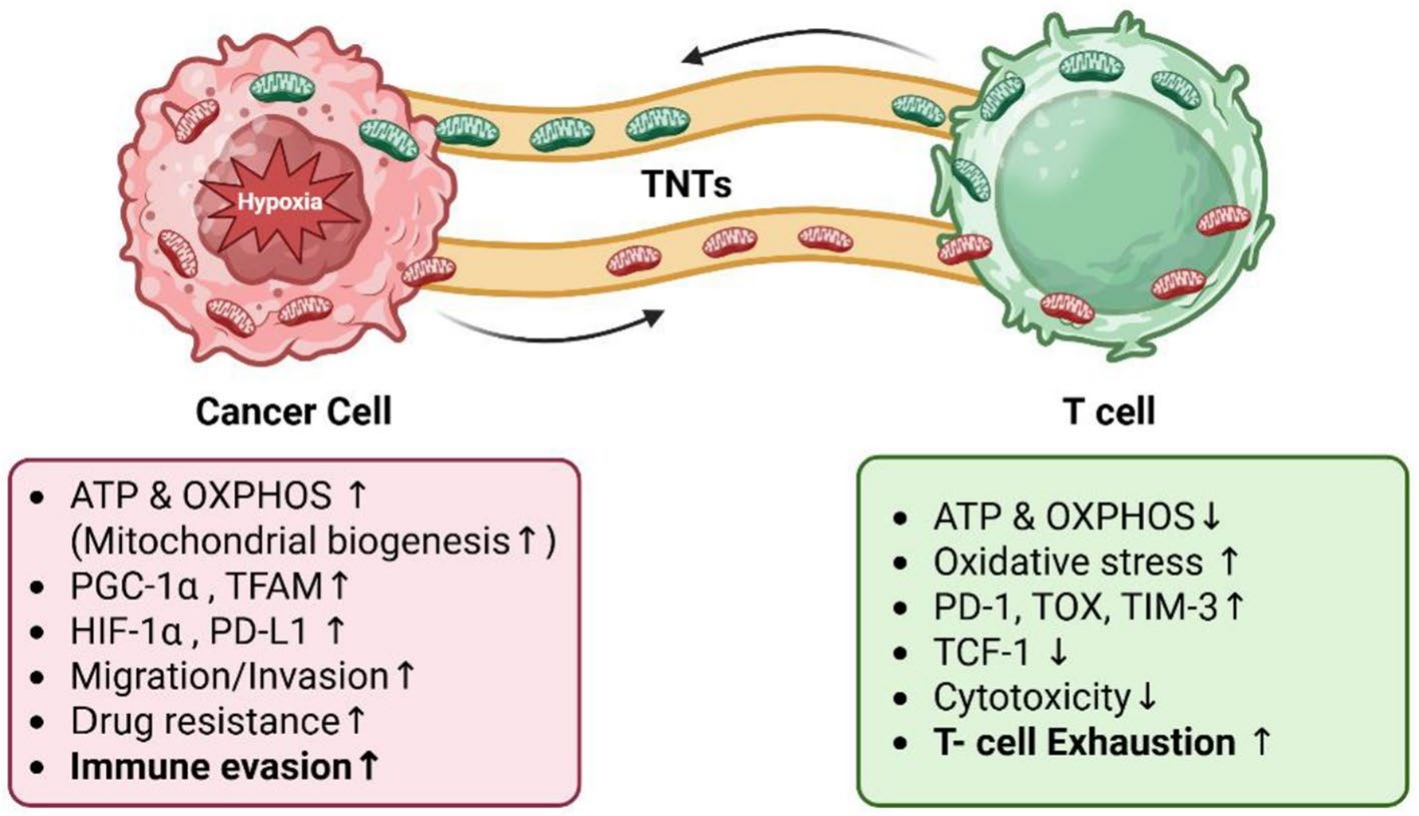
Metabolic and immunological consequences of mitochondrial transfer between T cells and tumor cells. This conceptual schematic illustrates TNT-mediated mitochondrial transfer between cancer cells and T cells. Mitochondria transferred from hypoxic cancer cells enhance OXPHOS and ATP production in tumor cells, promoting metabolic adaptation and survival, while inducing oxidative stress and exhaustion markers (PD-1, TOX, TIM-3) in T cells. Together, these changes link tumor metabolism to immune evasion and therapy resistance within the TME. (Red mitochondria: Cancer cell-derived; Green mitochondria: T cell-derived.)

MT acts as a powerful immunomodulatory tool in cancer by rewiring the metabolic function of immune cells in two main directions. Tumor cells exploit TNT structures to hijack mitochondria from cytotoxic lymphocytes, particularly CD8⁺ T cells and natural killer cells. Live cell imaging and electron microscopy have revealed that cancer cells establish actin-based intercellular tubes to siphon mitochondria directly from immune cells [4]. This transfer deprives the lymphocytes of mitochondrial respiration and spare capacity, weakens their cytotoxicity, and enhances tumor cell bioenergetics and proliferation. Recent evidence shows that tumor cells can actively deposit mitochondria into infiltrating T cells within the TME [5]. In human lung cancer and melanoma specimens, dysfunctional mitochondria of tumor origin were found inside tumor-infiltrating CD8⁺ T cells, where they evaded autophagic clearance and caused accumulation of reactive oxygen species (ROS) [48]. The oxidative stress and metabolic perturbation induced by these extrinsic mitochondria drives the T cells into a state of functional exhaustion, characterized by elevated inhibitory checkpoints (PD-1, TOX) and loss of effector cytokine production. Clinically, the presence of tumor-derived mitochondrial DNA inside T cells correlated with poorer responses to anti-PD-1 immunotherapy. This suggests that MT from cancer to immune cells acts as a direct metabolic sabotage, reprogramming T cells toward ROS accumulation and diminishing their proliferative and cytotoxic capacity. Similar effects have been observed in NK cells and CD4⁺ T cells, where tumor-derived mitochondria impair cytotoxicity and cytokine production. These transfers, occurring via tunneling nanotubes, extracellular vesicles, or cell fusion, alter energy metabolism in recipient cells, contributing to immune escape, tumor progression, and reduced immunotherapy efficacy [49]. Together, these studies highlight mitochondrial exchange as a bidirectional immunomodulatory strategy: tumors either drain critical mitochondria from immune cells or deliver compromised mitochondria to them, in both cases undermining immune attack. To illustrate these immunometabolic processes within the tumor microenvironment, a conceptual schematic model is presented (Fig. 2).

In addition, mitochondrial trafficking also influences innate immune components in the tumor microenvironment. Tumor-associated macrophages (TAMs), especially the pro-tumorigenic M2-polarized macrophages, can donate mitochondria to cancer cells [50]. In one model, M2-like macrophages with fragmented mitochondria transferred the organelles to breast cancer cells, which unexpectedly did not integrate into the host mitochondrial network and instead persisted as discrete ROS-generating units [51]. The localized ROS signals from the macrophage-donated mitochondria promoted cancer cell proliferation and invasiveness without necessarily boosting ATP production, indicating that MT can serve as a signaling mechanism—for example, by increasing mitochondrial ROS to activate pro-survival and pro-mitogenic pathways in tumor cells [52].

On the other hand, there is evidence that some stromal cells attempt an anti-tumor response via mitochondrial donation. A recent study found that osteocytes in bone transfer mitochondria to metastatic cancer cells arriving in the bone niche, triggering the cGAS/STING innate immune pathway in those cancer cells and leading to an antitumor immune response and suppressed growth of bone metastases [53]. When this MT was genetically blocked by deleting a mitochondrial membrane fusion protein Mfn2 in osteocytes, the cancer cells evaded immune detection and bone metastases progressed. This intriguing finding suggests that not all MT events favor the tumor; under certain conditions, organelle transfer can increase tumor immunogenicity.

Furthermore, MT can also influence macrophage polarization within the TME. For example, mitochondrial impairment in TAMs may drive a shift toward a pro-inflammatory M1-like state, potentially enhancing anti-tumor immune responses [54]. MT in the TME tends to support immune evasion by tumors either by intrinsically enhancing tumor cell fitness or extrinsically impairing the anti-tumor immune cells. Collectively, MT has evolved from a cell biological curiosity into a key immunological modulator in cancer. By reprogramming immune cell metabolism either through loss of their mitochondria or gain of aberrant ones, tumors create an immunosuppressive milieu that helps them escape immune destruction.

While the bidirectional nature of MT between tumor and immune/stromal cells is increasingly recognized, the regulatory determinants that dictate the direction of transfer remain unclear. Evidence suggests that donor–recipient polarity depends on the relative mitochondrial membrane potential, redox status, and cytoskeletal tension between interacting cells [3, 4]. For example, high-stress tumor cells with depolarized mitochondria tend to act as recipients, whereas metabolically active stromal or immune cells with hyperpolarized mitochondria function as donors.

However, this polarity is not static and can be reversed under conditions of chronic immune activation or oxidative stress. Under prolonged immune attack, tumor cells may export damaged mitochondria to macrophages or T cells, potentially triggering ROS accumulation and exhaustion phenotypes [55, 56]. These direction-dependent exchanges ultimately determine cell-fate outcomes—restoring oxidative metabolism in tumors while inducing senescence or dysfunction in immune cells. Thus, elucidating the molecular cues that reverse the direction of mitochondrial flow—such as MIRO1–RHOT1 signaling and redox-dependent TNT polarity—will be critical for developing selective MT-modulating therapies.

### Role in therapy resistance and tumor-specific adaptations

Another critical aspect of MT in cancer is its contribution to therapeutic resistance. Cancer treatments such as chemotherapy and radiation often induce mitochondrial damage or metabolic stress in tumor cells; mitochondrial exchange provides a route for stressed cancer cells to recover or circumvent these lethal insults [44, 57]. Several studies have shown that cancer cells can survive chemotherapy by importing mitochondria from supportive stromal cells, thus repairing their bioenergetic capacity. In acute myeloid leukemia (AML), for example, leukemic blasts under genotoxic stress stimulate bone marrow stromal cells to donate mitochondria through tunneling nanotubes, which in turn protect the leukemia cells during chemotherapy [58]. The transferred mitochondria in AML increase the leukemic cells’ oxidative phosphorylation activity and ATP levels, making them less dependent on glycolysis, a metabolic shift associated with resistance to cytotoxic drugs such as cytarabine [59]. In fact, inhibiting the MT process in AML significantly increased leukemic cell apoptosis and improved survival in mouse models, highlighting the critical role of organelle exchange in chemoresistance. This phenomenon is not limited to hematologic malignancies; in solid tumors, MT has also been linked to drug resistance. Endothelial cells preferentially transfer mitochondria to breast cancer cells via TNTs when the cancer cells are exposed to chemotherapeutic stress; this led to dramatically enhanced resistance of the cancer cells to doxorubicin [2]. The donated mitochondria help the cancer cells repair drug-induced mitochondrial damage and maintain energy production, thereby evading cell death. Mitochondrial trafficking has also been implicated in resistance of other hematologic malignancies and even rheumatologic diseases to treatment [60].

Although less studied than chemotherapy, emerging evidence suggests that MT may help tumor cells tolerate radiotherapy. Ionizing radiation can damage mitochondria and increase oxidative stress [61, 62]. It has been proposed that mitochondrial exchange from healthy cells could help replenish antioxidant capacity or repair oxidative phosphorylation in irradiated tumor cells. For instance, mesenchymal stromal cells co-cultured with irradiated breast cancer cells transferred mitochondria that reduced radiation-induced ROS in the cancer cells and improved their survival, similar to observations in chemotherapy-induced stress studies [63]. Additionally, horizontal transfer of mtDNA alone can restore respiratory function in tumor cells with radiation-damaged mitochondrial genomes [64]. By maintaining mitochondrial function, cancer cells are better able to manage radiation stress and may activate DNA repair pathways more effectively, contributing to radioresistance, although direct in vivo evidence for this is still an active area of research.

In summary, by exchanging mitochondria, cancer cells gain access to alternative sources of metabolic machinery that allow them to withstand lethal treatments. This has significant clinical implications: blocking intercellular MT could potentiate the efficacy of chemotherapy or targeted agents by removing a key resistance mechanism.

While the core mechanisms and effects of MT are shared, the extent and nuances of this phenomenon can vary across different cancers and microenvironments. Solid tumors, including lung, breast, prostate, brain, liver cancers often reside in heterogeneous, hypoxic environments that favor cell–cell interactions like TNT formation [65, 66]. MT frequently occurs between cancer cells and local stromal cells such as fibroblasts, endothelial cells, or tissue-specific support cells. In solid tumors, MT is often exploited for invasion. For instance, melanoma cells in the brain can acquire mitochondria from astrocytes to colonize the brain, and ovarian cancer cells in peritoneal metastases obtain mitochondria from mesothelial or endothelial cells to support outgrowth [67]. The significance of MT in solid tumors is underscored by its link to aggressive traits. A higher degree of intercellular mitochondrial exchange is associated with greater metastatic ability and therapy resistance in models of breast [68], ovarian [46], and prostate cancers [69].

By contrast, in hematologic malignancies such as leukemias and myelomas, the bone marrow niche provides a unique setting for MT. Here, bone marrow mesenchymal stromal cells serve as key donors of mitochondria to malignant cells. AML blasts form tunneling nanotubes or utilize macropinocytosis to import mitochondria from mesenchymal stromal cells, which is crucial for their survival under treatment [70, 71]. The close physical association between leukemia cells and marrow stromal cells, often involving direct contact at specialized niches, facilitates efficient organelle exchange.

It is noteworthy that not all cancer types utilize MT in the same way under therapy pressure. For example, AML primarily receives mitochondria [70], whereas T cell acute lymphoblastic leukemia (T-ALL) tends to donate mitochondria away under stress [72]. This reverse transfer from T-ALL to mesenchymal cells led to a reduction of ROS in the T-ALL cells since they offloaded damaged mitochondria, ultimately promoting T-ALL survival and drug resistance. The difference between AML and ALL is thought to arise from their distinct metabolic needs and cell adhesion properties. AML cells rely more on oxidative phosphorylation and readily import mitochondria, whereas T-ALL cells (more glycolytic) may instead export dysfunctional mitochondria to cope with stress [73]. Together, these findings underscore that MT is a flexible adaptive strategy, but with the result of enhancing tumor cell survival against therapy.

## Experimental approaches to study MT

### Methods to trace MT

Methods to trace intercellular MT now integrate live imaging, genetic reporters, natural mitochondrial barcodes, microfluidic pairing, and functional delivery assays [74–76]. Consensus guidance emphasizes genetic labeling, dye-independent readouts, orthogonal validation, and explicit reporting of negative and technical controls [77]. Genetically encoded mitochondrial reporters enable selective labeling of donor organelles and direct tracking of entry into recipient cells in vitro and in vivo. In vivo lineage tracing with mitochondria-targeted fluorophores has demonstrated host-derived mitochondrial signals inside implanted tumor cells in permissive microenvironments, confirming uptake in situ [45]. Fate mapping clarifies whether imported mitochondria persist, integrate, or are eliminated. In tumor models, transferred organelles can remain long-lived and reprogram recipient signaling, motivating the pairing of lineage evidence with mitophagy and functional reporters to distinguish persistence from degradation after transfer [52, 74].

Although dye-based approaches are widely used, they are prone to dye leakage, nonspecific uptake, and confounding from cell fusion or extracellular debris. Therefore, recent papers recommend against relying on dyes alone, and instead recommend validation using mitochondrial DNA haplotyping, donor-specific mitochondrial markers, genetically encoded reporters, and controls that exclude adhesion and phagocytosis [77].

High-throughput discovery is supported by droplet-based microfluidic systems that co-culture donor and recipient cells. These platforms quantify transfer frequency and link organelle exchange to downstream metabolic effects with precise control over pairing and dosage [78]. Recent advances in single-cell sequencing technologies have made it possible to analyze mitochondrial DNA (mtDNA) mutations. The MERCI pipeline, for instance, analyzes mtDNA single-nucleotide variants within single-cell RNA sequencing datasets to reconstruct donor-recipient relationships in human tumors. This method has been successfully applied to identify tumor-infiltrating T cells that have acquired tumor-derived mitochondria [79]. Complementary technologies enrich mitochondrial variants in single-cell RNA sequencing to recover clonal lineages at scale [80] and profile mitochondrial genotypes together with chromatin accessibility using mitochondrial single-cell ATAC-seq, enabling simultaneous lineage mapping and cell state readout [80–82]. A new computational method designed to identify informative mitochondrial mutations from single-cell sequencing data can also be used to track mitochondrial variation between cells [83].

Recently, genetic reporter systems such as Mito-TRACER have been introduced, which permanently label recipient cells that have taken up exogenous mitochondria [84]. These tools enable lineage tracing of mitochondrial transfer processes and have already provided valuable insights. With the widespread adoption and improvement of these reporters, single-cell sequencing approaches for detecting exogenous mtDNA within cells will significantly improve detection performance. Together, these complementary methods allow researchers to visualize mitochondrial movement, confirm their origin, and quantify their functional integration upon arrival. Integrating genetic reporters, natural barcodes, delivery readouts, nanotube-resolved imaging, and fate mapping yields a rigorous toolkit for mechanistic and translational studies of MT in cancer, with a premium on approaches validated in the recent literature from high impact venues [3, 79, 85].

### Technical challenges and future directions

One major challenge is reliably detecting and quantifying MT in vivo. Most current evidence relies on microscopy-based observations, which are prone to experimental artifacts. For instance, dye transfer can occur via gap junctions or released vesicles, creating false positives, and cell fusion or cell engulfment events can be misinterpreted as organelle transfer. Moreover, quantitative metrics are lacking; researchers cannot yet readily quantify how many mitochondria are transferred per cell or what fraction of cells engage in transfer in a tumor. Flow cytometry and high-resolution live imaging techniques are being developed to address this, but standardization is needed.

Another challenge is establishing causal links between MT and downstream functional changes such as immune cell exhaustion or drug resistance [86]. While correlative evidence is strong, functional assays need to directly tie the acquisition of mitochondria to specific phenotypic changes. Achieving this requires better spatiotemporal control in experimental systems, such as optogenetic or photoactivatable markers that can trigger or halt mitochondrial movement on demand. Future studies will likely integrate immune phenotyping, including T cell exhaustion markers like PD-1 and TOX, into MT experiments to map cause and effect more clearly [22].

At the molecular level, many questions remain about how cells recognize and handle transferred mitochondria. Do recipient cells treat incoming mitochondria differently? For instance, are they sequestered, actively integrated, or selectively degraded? Some studies suggest the transferred mitochondria can resist mitophagy and form a semi-autonomous population within the host cell [87]. Understanding the fate of donated mitochondria may reveal vulnerabilities. For example, if they depend on certain fission/fusion proteins (Mfn1/2, Drp1) or chaperones to become functional in the new host, those could be targeted. Genomic technologies will be valuable here: single-cell RNA sequencing (scRNA-seq) coupled with mitochondrial genotyping can identify cells that carry another cell’s mtDNA and reveal consequent changes in gene expression [22]. CRISPR-Cas9 screens focused on mitochondrial trafficking genes (e.g., MIRO1, MFN2, or TNT regulatory genes like ARPC3 or CDC42) in co-culture systems could pinpoint key regulators of intercellular MT. Screening in tri-culture models comprising tumor, stromal, and immune cells that recapitulate the TME may enable discovery of interventions that selectively inhibit pathological MT while minimizing off-target cytotoxicity.

Cutting-edge technologies are rapidly advancing to address current challenges in MT research. Single-cell metabolomics and spatial transcriptomics can map metabolic states and cell interactions in situ, potentially capturing MT events as they happen in tissue. For example, scRNA-seq combined with computational pipelines like MERCI enables quantitative assessment of mitochondrial DNA variant transfer between cell types in situ [88]. Artificial intelligence (AI)-based image analysis may help identify TNT structures or mitochondrial exchange patterns in pathology slides that are invisible to the human eye, uncovering clinical correlations [89]. These AI-powered platforms improve the reproducibility and throughput of microscopy analyses essential for biomarker discovery. Furthermore, innovative therapeutic delivery systems could be developed. For instance, nanoparticles or engineered vesicles that specifically target transferred mitochondria or the tunneling nanotube structures. One speculative but exciting notion is harnessing the MT mechanism itself as a Trojan horse – for example, loading a normal cell’s mitochondria with a therapeutic agent or sensor and allowing the tumor to take them up via TNTs [90].

Although the role of MT in cancer has been increasingly recognized, the current body of evidence remains heterogeneous and, in some cases, contradictory. In most tumor models, MT enhances oxidative phosphorylation and supports tumor survival by depleting mitochondrial reserves from cytotoxic lymphocytes [3, 4]. However, other studies demonstrate that the reverse process—from tumor to stromal or immune cells—can paradoxically activate innate immune pathways such as cGAS/STING signaling, thereby promoting antitumor immunity [53]. These divergent outcomes indicate that the biological consequences of MT are not uniform but depend critically on transfer directionality, donor–recipient identity, and the metabolic or functional state of the transferred mitochondria [5]. Collectively, these findings underscore that MT should be viewed as a context-dependent process shaped by microenvironmental and metabolic cues, rather than a universally tumor-promoting mechanism.

Beyond these conceptual inconsistencies, methodological limitations further complicate interpretation. Many studies rely on dye-based imaging or short-term co-culture assays that can overestimate or misinterpret organelle exchange events. In contrast, more rigorous approaches—such as genetic reporters [6] and single-cell mtDNA lineage tracing [79]—have confirmed MT in vivo but may underestimate transient or bidirectional exchanges. Quantitative frameworks for assessing MT frequency, duration, and persistence across heterogeneous tumor subregions remain underdeveloped.

To resolve these gaps, future studies should integrate single-cell multi-omics, spatial metabolomics, and real-time mitochondrial imaging to establish causal links between MT and downstream processes such as immune exhaustion, metabolic reprogramming, and therapy resistance. Ultimately, a standardized and context-aware experimental framework will be essential to advance MT research from descriptive observation to a reproducible and clinically actionable concept.

## Translational and therapeutic perspectives

### Emerging therapeutic strategies targeting MT

As research moves toward clinical application, investigators are seeking biomarkers of MT that could indicate which patients or tumors are highly dependent on this process. One idea is that high expression of mitochondrial trafficking proteins (such as MIRO1 or TNT-facilitating proteins) in a tumor biopsy could correlate with aggressive behavior or immune therapy resistance [29, 91]. Similarly, detecting hybrid mitochondrial populations (e.g., cancer cells containing mtDNA haplotypes from surrounding normal cells) in patient samples might serve as a marker of extensive mitochondrial exchange. These biomarkers could guide patient stratification. For example, patients with high MT activity might benefit from therapies that include an MT inhibitor or metabolic modulator.

Emerging therapies aim either to hamper the transfer conduits, neutralize upstream signaling cues, or enhance the resilience of recipient immune cells. One of the most direct strategies is to prevent the formation of TNTs. Disrupting the physical pathway can potentially block mitochondrial trafficking [56]. Approaches to prevent TNT structure formation include using pharmacological inhibitors of actin polymerization or targeting signaling pathways that induce TNT formation [18]. For instance, blocking inflammatory cytokines such as IL-6 and TNF-α, which promote TNT formation, can reduce the frequency of TNT expression [92]. Preclinical experiments have shown that treating cancer cell co-cultures with actin inhibitors such as cytochalasin B or latrunculin decreases organelle transfer and can increase tumor cell sensitivity to chemotherapy [1]. However, because general actin inhibitors have off-target effects, current research is exploring more specific TNT blockers. One promising target is CDC42—a small GTPase that regulates filopodia and TNT formation—as well as other members of the Rho family of GTPases. Inhibition of these GTPases has been shown to decrease mitochondrial exchange between cells in vitro [93–95]. Another target is Miro1, a mitochondrial trafficking protein on the outer mitochondrial membrane that facilitates organelle movement. Downregulating or inhibiting Miro1 in donor cells can prevent efficient mitochondrial delivery to tumor cells [91].

In addition, strategies to interfere with MT via extracellular vesicles are also under consideration. These could include inhibitors of exosome release (such as GW4869, which blocks neutral sphingomyelinase and exosome biogenesis) to reduce the secretion of mtDNA-laden vesicles from tumor or stromal cells [96]. Another strategy is to block uptake pathways in recipient cells. Inhibiting macropinocytosis or endocytosis can reduce the internalization of vesicles that carry mitochondria [97]. Representative molecular targets, inhibitors, and levels of supporting evidence are summarized to provide an overview of MT-modulating strategies (Table 1).

**Table 1:** Proposed Therapeutic Target of TNT-mediated MT

Target to inhibit	Mechanism of Action	Related Inhibitor	Key References
*RAC1*	Blocks actin dynamics and TNT formation, Reduced mitochondrial transfer	NSC23766, EHT1864, ZINC69391	[109–113]
*RHOT1* *(Miro1)*	Disrupts mitochondrial trafficking and TNT function, Impaired tumor adaptation to hypoxia, reduced cancer survival	**Miro1 Reducer** (CAS 2624336–91-8)	[25, 113, 114]
*MTFR2*	Limits mitochondrial fission and transfer, suppresses tumor growth	None	[39–41, 115, 116]
*DRP1*	Inhibits mitochondrial fission, reduces mitochondrial transfer	Mdivi-1, MIDI, P110, PPD1	[36, 117–119]

In the immunotherapy setting, preserving T cell function has become a major goal, since tumor-derived mitochondria are increasingly implicated in driving T cell exhaustion [98]. Metabolic adjuvants such as low-dose metformin have been shown to strengthen T cell memory and counteract oxidative reprogramming. Early-phase studies combining metabolic modulators (e.g., metformin) with immune checkpoint inhibitors have shown potential to restore T-cell metabolism and counteract MT-driven exhaustion [98–100]. Additionally, mitochondrial augmentation of cytotoxic T cells before adoptive transfer has been shown to increase respiratory reserve and enhance resilience against tumor-induced mitochondrial depletion [101].

Targeting upstream triggers of transfer provides a complementary strategy. In AML, NADPH oxidase 2 was shown to initiate mitochondrial donation from stromal cells, and its pharmacological inhibition not only blocked transfer but also sensitized leukemic cells to chemotherapy [73, 102]. Other microenvironmental signals, including reactive oxygen species, calcium flux, and mechano-transduction pathways, are being examined as drivers of mitochondrial trafficking. The emerging concept of ‘mitokines,’ soluble factors released in response to mitochondrial stress that stimulate organelle donation, presents additional opportunities for therapeutic intervention once such mediators are identified [103].

Because MT frequently underlies resistance to cytotoxic and immune therapies, combination approaches are particularly attractive. Inhibiting TNTs or EV-mediated pathways alongside chemotherapy could prevent metabolic rescue of tumor cells by stromal support. In immuno-oncology, blocking mitochondrial hijacking in conjunction with immune checkpoint blockade may preserve T cell function, thereby improving treatment efficacy [104].

At present, no approved therapies specifically inhibit intercellular MT, and careful consideration is required to avoid interfering with physiological mitochondrial exchange that supports wound healing, immune surveillance, and tissue regeneration.

### Feasibility and safety considerations

Although TNT inhibition holds therapeutic promise in cancer, it also raises safety concerns because TNTs play essential roles in normal physiology. For instance, mitochondrial transfer from mesenchymal stem cells to injured alveolar epithelial cells promotes tissue repair [105], and astrocyte-to-neuron mitochondrial donation via TNTs supports neuronal recovery after ischemic injury [106]. In immune systems, TNTs facilitate antigen transfer and Ca^2^⁺-dependent communication between dendritic and T cells [107]. Hence, non-selective TNT blockade—through global inhibition of actin polymerization or GTPase activity—could impair regeneration and immune homeostasis. Future therapeutic approaches should therefore focus on tumor-specific molecular mediators such as MIRO1 or TNFAIP2, and employ localized or transient inhibition strategies [108]. Such selective modulation may maximize antitumor efficacy while minimizing off-target effects on physiological tissue repair.

## Conclusion

In recent years, intercellular MT has emerged as a key mechanism in cancer biology, transcending mere intracellular specificity. No longer confined to their role of intracellular powerhouses, mitochondria have become mobile organelles, crossing cell boundaries and reshaping the TME. Through this exchange, cancer cells acquire strategic advantages in survival, adaptation, and immune evasion. They restore damaged bioenergetics, gain resistance to stressors, escape immune surveillance, and adapt more flexibly to therapeutic pressures. Whether they acquire functional mitochondria from stromal or immune cells or remove damaged organelles, tumors utilize mitochondrial transport to their advantage. These insights into MT bring with them both promise and complexity. The prospect of targeting MT as a therapeutic strategy is compelling: interrupting these intercellular exchanges could destabilize tumor metabolism or restore immune cell competence. However, such interventions require caution, as MT also occurs in normal physiological contexts such as tissue repair and immune modulation.

MT redefines how we perceive tumor-host cell communication, introducing a new dimension of metabolic interdependence. This dynamic exchange not only enriches our understanding of cancer progression but also uncovers new therapeutic vulnerabilities. As research advances, the goal will be to selectively interrupt these metabolic processes in tumors while preserving physiological roles elsewhere. With precision tools and collaborative experimental methods becoming available, MT may soon become a cornerstone of metabolic oncology.

## Data Availability

No datasets were generated or analysed during the current study.

## Abbreviations

ATP: Adenosine triphosphate
CAF: Cancer-associated fibroblast
EV: Extracellular Vesicles
HCC: Hepatocellular carcinoma
MT: Mitochondrial transfer
mtDNA: Mitochondrial DNA
OXPHOS: Oxidative phosphorylation
scRNA-seq: Single-Cell RNA Sequencing
PD-1: Programmed cell death protein 1
ROS: Reactive oxygen species
TIL: Tumor-Infiltrating Lymphocytes
TME: Tumor microenvironment
TNTs: Tunneling nanotubes

## References

[1] Guan F, Wu X, Zhou J, Lin Y, He Y, Fan C, et al. Mitochondrial transfer in tunneling nanotubes-a new target for cancer therapy. J Exp Clin Cancer Res. 2024;43(1):147. 38769583 10.1186/s13046-024-03069-w PMC11106947

[2] Pasquier J, Guerrouahen BS, Al Thawadi H, Ghiabi P, Maleki M, Abu-Kaoud N, et al. Preferential transfer of mitochondria from endothelial to cancer cells through tunneling nanotubes modulates chemoresistance. J Transl Med. 2013;11:94. 23574623 10.1186/1479-5876-11-94 PMC3668949

[3] Saha T, Dash C, Jayabalan R, Khiste S, Kulkarni A, Kurmi K, et al. Intercellular nanotubes mediate mitochondrial trafficking between cancer and immune cells. Nat Nanotechnol. 2022;17(1):98–106. 34795441 10.1038/s41565-021-01000-4 PMC10071558

[4] Ikeda H, Kawase K, Nishi T, Watanabe T, Takenaga K, Inozume T, et al. Immune evasion through mitochondrial transfer in the tumour microenvironment. Nature. 2025;638(8049):225–36. 39843734 10.1038/s41586-024-08439-0 PMC11798832

[5] Baldwin JG, Heuser-Loy C, Saha T, Schelker RC, Slavkovic-Lukic D, Strieder N, et al. Intercellular nanotube-mediated mitochondrial transfer enhances T cell metabolic fitness and antitumor efficacy. Cell. 2024;187(23):6614-30.e21. 39276774 10.1016/j.cell.2024.08.029 PMC11623344

[6] de Mello NP, Fecher C, Pastor AM, Perocchi F, Misgeld T. Ex vivo immunocapture and functional characterization of cell-type-specific mitochondria using MitoTag mice. Nat Protoc. 2023;18(7):2181–220. 37328604 10.1038/s41596-023-00831-w

[7] Nitsch L, Lareau CA, Ludwig LS. Mitochondrial genetics through the lens of single-cell multi-omics. Nat Genet. 2024;56(7):1355–65. 38951641 10.1038/s41588-024-01794-8 PMC11260401

[8] Du H, Xu T, Yu S, Wu S, Zhang J. Mitochondrial metabolism and cancer therapeutic innovation. Signal Transduct Target Ther. 2025;10(1):245. 40754534 10.1038/s41392-025-02311-x PMC12319113

[9] Barutta F, Bellini S, Kimura S, Hase K, Corbetta B, Corbelli A, et al. Protective effect of the tunneling nanotube-TNFAIP2/M-sec system on podocyte autophagy in diabetic nephropathy. Autophagy. 2023;19(2):505–24. 35659195 10.1080/15548627.2022.2080382 PMC9851239

[10] Chen Y, Xiao D, Li X. The role of mitochondrial transfer via tunneling nanotubes in the central nervous system: a review. Medicine (Baltimore). 2024;103(9):e37352. 38428884 10.1097/MD.0000000000037352 PMC10906627

[11] Leonov S, Dorfman A, Pershikova E, Inyang O, Alhaddad L, Wang Y, et al. Extracellular vesicle- and mitochondria-based targeting of non-small cell lung cancer response to radiation: challenges and perspectives. Cancers (Basel). 2024;16(12):2235. 38927940 10.3390/cancers16122235 PMC11201585

[12] Zhao L, Liu P, Kepp O, Kroemer G. Mitochondrial DNA transfer between malignant cells and T lymphocytes shapes the cancer-immunity dialogue. Oncoimmunology. 2025;14(1):2512109. 40434021 10.1080/2162402X.2025.2512109 PMC12123971

[13] Changaei M, Azimzadeh Tabrizi Z, Karimi M, Kashfi SA, Koochaki Chahardeh T, Hashemi SM, et al. From powerhouse to modulator: regulating immune system responses through intracellular mitochondrial transfer. Cell Commun Signal. 2025;23(1):232. 40394666 10.1186/s12964-025-02237-5 PMC12090700

[14] Jing M, Xiong X, Mao X, Song Q, Zhang L, Ouyang Y, et al. HMGB1 promotes mitochondrial transfer between hepatocellular carcinoma cells through RHOT1 and RAC1 under hypoxia. Cell Death Dis. 2024;15(2):155. 38378644 10.1038/s41419-024-06536-6 PMC10879213

[15] Kong J, Sun R, Du C, Tang Y, Xie C, Li Q, et al. Mitochondrial extracellular vesicles: a novel approach to mitochondrial quality control. Biomolecules. 2025;15(8):1145. 40867590 10.3390/biom15081145 PMC12384377

[16] Carles-Fontana R, Heaton N, Palma E, Khorsandi SE. Extracellular vesicle-mediated mitochondrial reprogramming in cancer. Cancers (Basel). 2022;14(8):1865. 35454774 10.3390/cancers14081865 PMC9032679

[17] Iorio R, Petricca S, Di Emidio G, Falone S, Tatone C. Mitochondrial extracellular vesicles (mitoEVs): emerging mediators of cell-to-cell communication in health, aging and age-related diseases. Ageing Res Rev. 2024;101:102522. 39369800 10.1016/j.arr.2024.102522

[18] Peng X, Gao Y, Liu J, Shi X, Li W, Ma Y, et al. Mitochondria-derived vesicles: a promising and potential target for tumour therapy. Clin Transl Med. 2025;15(5):e70320. 40356246 10.1002/ctm2.70320 PMC12069804

[19] Qiao X, Huang N, Meng W, Liu Y, Li J, Li C, et al. Beyond mitochondrial transfer, cell fusion rescues metabolic dysfunction and boosts malignancy in adenoid cystic carcinoma. Cell Rep. 2024;43(9):114652. 39217612 10.1016/j.celrep.2024.114652

[20] Gervasi A, D’Aprile S, Denaro S, Amorini MA, Vicario N, Parenti R. Connexin 43 role in mitochondrial transfer and homeostasis in the central nervous system. J Cell Physiol. 2025;240(8):e70086. 40838510 10.1002/jcp.70086 PMC12368938

[21] Fu H, Xie X, Zhai L, Liu Y, Tang Y, He S, et al. CX43-mediated mitochondrial transfer maintains stemness of KG-1a leukemia stem cells through metabolic remodeling. Stem Cell Res Ther. 2024;15(1):460. 39623456 10.1186/s13287-024-04079-3 PMC11613858

[22] Liu R, Shan W, Wang Z, Wang H, Li C, Yang L, et al. Unveiling mitochondrial transfer in tumor immune evasion: mechanisms, challenges, and clinical implications. Front Immunol. 2025;16:1625814. 40766302 10.3389/fimmu.2025.1625814 PMC12321546

[23] Zampieri LX, Silva-Almeida C, Rondeau JD, Sonveaux P. Mitochondrial transfer in cancer: a comprehensive review. Int J Mol Sci. 2021;22(6):3245. 33806730 10.3390/ijms22063245 PMC8004668

[24] Sahinbegovic H, Jelinek T, Hrdinka M, Bago JR, Turi M, Sevcikova T, et al. Intercellular mitochondrial transfer in the tumor microenvironment. Cancers (Basel). 2020;12(7):1787. 32635428 10.3390/cancers12071787 PMC7407231

[25] Nahacka Z, Novak J, Zobalova R, Neuzil J. Miro proteins and their role in mitochondrial transfer in cancer and beyond. Front Cell Dev Biol. 2022;10:937753. 35959487 10.3389/fcell.2022.937753 PMC9358137

[26] Lopez-Domenech G, Covill-Cooke C, Ivankovic D, Halff EF, Sheehan DF, Norkett R, et al. Miro proteins coordinate microtubule- and actin-dependent mitochondrial transport and distribution. EMBO J. 2018;37(3):321–36. 29311115 10.15252/embj.201696380 PMC5793800

[27] Las G, Shirihai OS. Miro1: new wheels for transferring mitochondria. EMBO J. 2014;33(9):939–41. 24711517 10.1002/embj.201488441 PMC4193928

[28] Alshaabi H, Shannon N, Gravelle R, Milczarek S, Messier T, Cunniff B. Miro1-mediated mitochondrial positioning supports subcellular redox status. Redox Biol. 2021;38:101818. 33341544 10.1016/j.redox.2020.101818 PMC7753203

[29] Boulton DP, Caino MC. Emerging roles for mitochondrial Rho GTPases in tumor biology. J Biol Chem. 2024;300(9):107670. 39128718 10.1016/j.jbc.2024.107670 PMC11402688

[30] Cangkrama M, Liu H, Whipman J, Zubair M, Matsushita M, Di Filippo M, et al. A protumorigenic mDia2-MIRO1 Axis controls mitochondrial positioning and function in cancer-associated fibroblasts. Cancer Res. 2022;82(20):3701–17. 35997559 10.1158/0008-5472.CAN-22-0162 PMC9574377

[31] Babenko VA, Silachev DN, Popkov VA, Zorova LD, Pevzner IB, Plotnikov EY, et al. Miro1 enhances mitochondria transfer from multipotent mesenchymal stem cells (MMSC) to neural cells and improves the efficacy of cell recovery. Molecules. 2018;23(3):687. 29562677 10.3390/molecules23030687 PMC6017474

[32] Zhan L, Cao H, Wang G, Lyu Y, Sun X, An J, et al. Drp1-mediated mitochondrial fission promotes cell proliferation through crosstalk of p53 and NF-kappaB pathways in hepatocellular carcinoma. Oncotarget. 2016;7(40):65001–11. 27542250 10.18632/oncotarget.11339 PMC5323133

[33] Wang N, Wang X, Lan B, Gao Y, Cai Y. DRP1, fission and apoptosis. Cell Death Discov. 2025;11(1):150. 40195359 10.1038/s41420-025-02458-0 PMC11977278

[34] Zerihun M, Sukumaran S, Qvit N. The Drp1-mediated mitochondrial fission protein interactome as an emerging core player in mitochondrial dynamics and cardiovascular disease therapy. Int J Mol Sci. 2023;24(6):5785. 36982862 10.3390/ijms24065785 PMC10057413

[35] Horn SR, Thomenius MJ, Johnson ES, Freel CD, Wu JQ, Coloff JL, et al. Regulation of mitochondrial morphology by APC/CCdh1-mediated control of Drp1 stability. Mol Biol Cell. 2011;22(8):1207–16. 21325626 10.1091/mbc.E10-07-0567 PMC3078078

[36] Lin XH, Qiu BQ, Ma M, Zhang R, Hsu SJ, Liu HH, et al. Suppressing DRP1-mediated mitochondrial fission and mitophagy increases mitochondrial apoptosis of hepatocellular carcinoma cells in the setting of hypoxia. Oncogenesis. 2020;9(7):67. 32661251 10.1038/s41389-020-00251-5 PMC7359348

[37] Bao D, Zhao J, Zhou X, Yang Q, Chen Y, Zhu J, et al. Mitochondrial fission-induced mtDNA stress promotes tumor-associated macrophage infiltration and HCC progression. Oncogene. 2019;38(25):5007–20. 30894684 10.1038/s41388-019-0772-z PMC6755992

[38] Bian J, Zhang D, Wang Y, Qin H, Yang W, Cui R, et al. Mitochondrial quality control in hepatocellular carcinoma. Front Oncol. 2021;11:713721. 34589426 10.3389/fonc.2021.713721 PMC8473831

[39] Zhang L, Zhang X, Liu H, Yang C, Yu J, Zhao W, et al. MTFR2-dependent mitochondrial fission promotes HCC progression. J Transl Med. 2024;22(1):73. 38238834 10.1186/s12967-023-04845-6 PMC10795309

[40] Zhang L, Zhou B, Yang J, Ren C, Luo J, Li Z, et al. MTFR2-mediated fission drives fatty acid and mitochondrial co-transfer from hepatic stellate cells to tumor cells fueling oncogenesis. Adv Sci. 2025;12(23):e2416419. 10.1002/advs.202416419 PMC12199435 40365837

[41] Huang Q, Han Y, Shen E, Feng Z, Peng Y, Gao L, et al. MTFR2 shapes a barrier of immune microenvironment in hepatocellular carcinoma. iScience. 2023;26(1):105095. 36713263 10.1016/j.isci.2022.105095 PMC9881049

[42] Lee HY, Nga HT, Tian J, Yi HS. Mitochondrial metabolic signatures in hepatocellular carcinoma. Cells. 2021;10(8):1901. 34440674 10.3390/cells10081901 PMC8391498

[43] Wu Z, Xiao C, Li F, Huang W, You F, Li X. Mitochondrial fusion-fission dynamics and its involvement in colorectal cancer. Mol Oncol. 2024;18(5):1058–75. 38158734 10.1002/1878-0261.13578 PMC11076987

[44] Marabitti V, Vulpis E, Nazio F, Campello S. Mitochondrial transfer as a strategy for enhancing cancer cell fitness: current insights and future directions. Pharmacol Res. 2024;208:107382. 39218420 10.1016/j.phrs.2024.107382

[45] Watson DC, Bayik D, Storevik S, Moreino SS, Sprowls SA, Han J, et al. GAP43-dependent mitochondria transfer from astrocytes enhances glioblastoma tumorigenicity. Nat Cancer. 2023;4(5):648–64. 37169842 10.1038/s43018-023-00556-5 PMC10212766

[46] Frisbie L, Pressimone C, Dyer E, Baruwal R, Garcia G, St Croix C, et al. Carcinoma-associated mesenchymal stem cells promote ovarian cancer heterogeneity and metastasis through mitochondrial transfer. Cell Rep. 2024;43(8):114551. 39067022 10.1016/j.celrep.2024.114551 PMC11420855

[47] Cangkrama M, Liu H, Wu X, Yates J, Whipman J, Gabelein CG, et al. MIRO2-mediated mitochondrial transfer from cancer cells induces cancer-associated fibroblast differentiation. Nat Cancer. 2025. 10.1038/s43018-025-01038-6. 40877413 10.1038/s43018-025-01038-6 PMC12559006

[48] Yang MQ, Zhang SL, Sun L, Huang LT, Yu J, Zhang JH, et al. Targeting mitochondria: restoring the antitumor efficacy of exhausted T cells. Mol Cancer. 2024;23(1):260. 39563438 10.1186/s12943-024-02175-9 PMC11575104

[49] Akhter W, Nakhle J, Vaillant L, Garcin G, Le Saout C, Simon M, et al. Transfer of mesenchymal stem cell mitochondria to CD4(+) T cells contributes to repress Th1 differentiation by downregulating T-bet expression. Stem Cell Res Ther. 2023;14(1):12. 36694226 10.1186/s13287-022-03219-x PMC9875419

[50] Bai R, Cui J. Mitochondrial immune regulation and anti-tumor immunotherapy strategies targeting mitochondria. Cancer Lett. 2023;564:216223. 37172686 10.1016/j.canlet.2023.216223

[51] Wang S, Wang J, Chen Z, Luo J, Guo W, Sun L, et al. Targeting M2-like tumor-associated macrophages is a potential therapeutic approach to overcome antitumor drug resistance. NPJ Precis Oncol. 2024;8(1):31. 38341519 10.1038/s41698-024-00522-z PMC10858952

[52] Kidwell CU, Casalini JR, Pradeep S, Scherer SD, Greiner D, Bayik D, et al. Transferred mitochondria accumulate reactive oxygen species, promoting proliferation. Elife. 2023;12:e85494. 36876914 10.7554/eLife.85494 PMC10042539

[53] Zhou H, Zhang W, Li H, Xu F, Yinwang E, Xue Y, et al. Osteocyte mitochondria inhibit tumor development via STING-dependent antitumor immunity. Sci Adv. 2024;10(3):eadi4298. 38232158 10.1126/sciadv.adi4298 PMC10793952

[54] Kiss M, Pittet MJ. Mitochondrial control of macrophage polarity in tumors. Immunity. 2025;58(7):1618–20. 40633525 10.1016/j.immuni.2025.06.008

[55] Kuo CL, Ponneri Babuharisankar A, Lin YC, Lien HW, Lo YK, Chou HY, et al. Mitochondrial oxidative stress in the tumor microenvironment and cancer immunoescape: foe or friend? J Biomed Sci. 2022;29(1):74. 36154922 10.1186/s12929-022-00859-2 PMC9511749

[56] Chun S, An J, Kim MS. Mitochondrial transfer between cancer and T cells: implications for immune evasion. Antioxidants. 2025;14(8):1008. 40867904 10.3390/antiox14081008 PMC12382691

[57] Bahar E, Han SY, Kim JY, Yoon H. Chemotherapy resistance: role of mitochondrial and autophagic components. Cancers (Basel). 2022;14(6):1462. 35326612 10.3390/cancers14061462 PMC8945922

[58] Tjahjono E, Daneman MR, Meika B, Revtovich AV, Kirienko NV. Mitochondrial abnormalities as a target of intervention in acute myeloid leukemia. Front Oncol. 2024;14:1532857. 39902131 10.3389/fonc.2024.1532857 PMC11788353

[59] Peruzzo R, Szabo I. Contribution of mitochondrial ion channels to chemo-resistance in cancer cells. Cancers (Basel). 2019;11(6):761. 31159324 10.3390/cancers11060761 PMC6627730

[60] Jin P, Jiang J, Zhou L, Huang Z, Nice EC, Huang C, et al. Mitochondrial adaptation in cancer drug resistance: prevalence, mechanisms, and management. J Hematol Oncol. 2022;15(1):97. 35851420 10.1186/s13045-022-01313-4 PMC9290242

[61] Wei Y, Xiao G, Xu H, Sun X, Shi Y, Wang F, et al. Radiation resistance of cancer cells caused by mitochondrial dysfunction depends on SIRT3-mediated mitophagy. FEBS J. 2023;290(14):3629–45. 36871142 10.1111/febs.16769

[62] Dong S, Lyu X, Yuan S, Wang S, Li W, Chen Z, et al. Oxidative stress: a critical hint in ionizing radiation induced pyroptosis. Radiat Med Prot. 2020;1(4):179–85.

[63] Ohkouchi S, Block GJ, Katsha AM, Kanehira M, Ebina M, Kikuchi T, et al. Mesenchymal stromal cells protect cancer cells from ROS-induced apoptosis and enhance the Warburg effect by secreting STC1. Mol Ther. 2012;20(2):417–23. 22146344 10.1038/mt.2011.259 PMC3277221

[64] Dong LF, Kovarova J, Bajzikova M, Bezawork-Geleta A, Svec D, Endaya B, et al. Horizontal transfer of whole mitochondria restores tumorigenic potential in mitochondrial DNA-deficient cancer cells. Elife. 2017;6:e22187. 28195532 10.7554/eLife.22187 PMC5367896

[65] Roehlecke C, Schmidt MHH. Tunneling nanotubes and tumor microtubes in cancer. Cancers (Basel). 2020;12(4):857. 32244839 10.3390/cancers12040857 PMC7226329

[66] Desir S, Dickson EL, Vogel RI, Thayanithy V, Wong P, Teoh D, et al. Tunneling nanotube formation is stimulated by hypoxia in ovarian cancer cells. Oncotarget. 2016;7(28):43150–61. 27223082 10.18632/oncotarget.9504 PMC5190014

[67] Kuo FC, Tsai HY, Cheng BL, Tsai KJ, Chen PC, Huang YB, et al. Endothelial mitochondria transfer to melanoma induces M2-type macrophage polarization and promotes tumor growth by the Nrf2/HO-1-mediated pathway. Int J Mol Sci. 2024;25(3):1857. 38339136 10.3390/ijms25031857 PMC10855867

[68] Libring S, Berestesky ED, Reinhart-King CA. The movement of mitochondria in breast cancer: internal motility and intercellular transfer of mitochondria. Clin Exp Metastasis. 2024;41(5):567–87. 38489056 10.1007/s10585-024-10269-3 PMC11499424

[69] Fontana F, Anselmi M, Limonta P. Unraveling the peculiar features of mitochondrial metabolism and dynamics in prostate cancer. Cancers (Basel). 2023;15(4):1192. 36831534 10.3390/cancers15041192 PMC9953833

[70] Moschoi R, Imbert V, Nebout M, Chiche J, Mary D, Prebet T, et al. Protective mitochondrial transfer from bone marrow stromal cells to acute myeloid leukemic cells during chemotherapy. Blood. 2016;128(2):253–64. 27257182 10.1182/blood-2015-07-655860

[71] Forte D, Garcia-Fernandez M, Sanchez-Aguilera A, Stavropoulou V, Fielding C, Martin-Perez D, et al. Bone marrow mesenchymal stem cells support acute myeloid leukemia bioenergetics and enhance antioxidant defense and escape from chemotherapy. Cell Metab. 2020;32(5):829-43.e9. 32966766 10.1016/j.cmet.2020.09.001 PMC7658808

[72] Wang J, Liu X, Qiu Y, Shi Y, Cai J, Wang B, et al. Cell adhesion-mediated mitochondria transfer contributes to mesenchymal stem cell-induced chemoresistance on T cell acute lymphoblastic leukemia cells. J Hematol Oncol. 2018;11(1):11. 29357914 10.1186/s13045-018-0554-z PMC5778754

[73] Guo X, Can C, Liu W, Wei Y, Yang X, Liu J, et al. Mitochondrial transfer in hematological malignancies. Biomark Res. 2023;11(1):89. 37798791 10.1186/s40364-023-00529-x PMC10557299

[74] Tiash S, Brestoff JR, Crewe C. A guide to studying mitochondria transfer. Nat Cell Biol. 2023;25(11):1551–3. 37853133 10.1038/s41556-023-01246-1 PMC11610514

[75] Qin Y, Jiang X, Yang Q, Zhao J, Zhou Q, Zhou Y. The functions, methods, and mobility of mitochondrial transfer between cells. Front Oncol. 2021;11:672781. 34041035 10.3389/fonc.2021.672781 PMC8141658

[76] Rusk N. Mitochondrial barcodes. Nat Methods. 2019;16(5):361. 31040429 10.1038/s41592-019-0416-9

[77] Brestoff JR, Singh KK, Aquilano K, Becker LB, Berridge MV, Boilard E, et al. Recommendations for mitochondria transfer and transplantation nomenclature and characterization. Nat Metab. 2025;7(1):53–67. 39820558 10.1038/s42255-024-01200-x

[78] Sun J, Lo HTJ, Fan L, Yiu TL, Shakoor A, Li G, et al. High-efficiency quantitative control of mitochondrial transfer based on droplet microfluidics and its application on muscle regeneration. Sci Adv. 2022;8(33):eabp9245. 35977014 10.1126/sciadv.abp9245 PMC9385153

[79] Zhang H, Yu X, Ye J, Li H, Hu J, Tan Y, et al. Systematic investigation of mitochondrial transfer between cancer cells and T cells at single-cell resolution. Cancer Cell. 2023;41(10):1788-802.e10. 37816332 10.1016/j.ccell.2023.09.003 PMC10568073

[80] Miller TE, Lareau CA, Verga JA, DePasquale EAK, Liu V, Ssozi D, et al. Mitochondrial variant enrichment from high-throughput single-cell RNA sequencing resolves clonal populations. Nat Biotechnol. 2022;40(7):1030–4. 35210612 10.1038/s41587-022-01210-8 PMC9288977

[81] Lareau CA, Liu V, Muus C, Praktiknjo SD, Nitsch L, Kautz P, et al. Mitochondrial single-cell ATAC-seq for high-throughput multi-omic detection of mitochondrial genotypes and chromatin accessibility. Nat Protoc. 2023;18(5):1416–40. 36792778 10.1038/s41596-022-00795-3 PMC10317201

[82] Kwok AWC, Qiao C, Huang R, Sham MH, Ho JWK, Huang Y. MQuad enables clonal substructure discovery using single cell mitochondrial variants. Nat Commun. 2022;13(1):1205. 35260582 10.1038/s41467-022-28845-0 PMC8904442

[83] Yu X, Hu J, Tan Y, Pan M, Zhang H, Li B. MitoTracer facilitates the identification of informative mitochondrial mutations for precise lineage reconstruction. PLoS Comput Biol. 2025;21(6):e1013090. 40549685 10.1371/journal.pcbi.1013090 PMC12184895

[84] Hoover G, Gilbert S, Curley O, Obellianne C, Lin MT, Hixson W, et al. Nerve-to-cancer transfer of mitochondria during cancer metastasis. Nature. 2025;644(8075):252–62. 40562940 10.1038/s41586-025-09176-8 PMC12328229

[85] Penter L, Ten Hacken E, Southard J, Lareau CA, Ludwig LS, Li S, et al. Mitochondrial DNA mutations as natural barcodes for lineage tracing of murine tumor models. Cancer Res. 2023;83(5):667–72. 36469010 10.1158/0008-5472.CAN-22-0275 PMC9988704

[86] Liu Y, Dissanayaka WL, Yiu C. Therapeutic implications of mitochondrial transfer on stem cell fate in regenerative medicine. J Transl Med. 2025;23(1):568. 40399970 10.1186/s12967-025-06472-9 PMC12093763

[87] Chen R, Chen J. Mitochondrial transfer - a novel promising approach for the treatment of metabolic diseases. Front Endocrinol (Lausanne). 2023;14:1346441. 38313834 10.3389/fendo.2023.1346441 PMC10837849

[88] Wu M, Pu W, Gu Z. Single-cell sequencing traces mitochondrial transfers. Genomics Proteomics Bioinformatics. 2025;22(6):qzae092. 39724171 10.1093/gpbjnl/qzae092 PMC11806949

[89] Ceran Y, Erguder H, Ladner K, Korenfeld S, Deniz K, Padmanabhan S, et al. TNTdetect. AI: a deep learning model for automated detection and counting of tunneling nanotubes in microscopy images. Cancers (Basel). 2022;14(19):4958. 36230881 10.3390/cancers14194958 PMC9562025

[90] Chen M, Zhao D. Invisible bridges: unveiling the role and prospects of tunneling nanotubes in cancer therapy. Mol Pharm. 2024;21(11):5413–29. 39373242 10.1021/acs.molpharmaceut.4c00563 PMC11539062

[91] Novak J, Nahacka Z, Oliveira GL, Brisudova P, Dubisova M, Dvorakova S, et al. The adaptor protein Miro1 modulates horizontal transfer of mitochondria in mouse melanoma models. Cell Rep. 2025;44(1):115154. 39792553 10.1016/j.celrep.2024.115154

[92] Yan L, Zheng D, Xu RH. Critical role of tumor necrosis factor signaling in mesenchymal stem cell-based therapy for autoimmune and inflammatory diseases. Front Immunol. 2018;9:1658. 30079066 10.3389/fimmu.2018.01658 PMC6062591

[93] Liu J, Guo H, Liu S, Hu Y, Huang Y, Rong J, et al. Blocking secretion of exosomes by GW4869 dampens CD8(+) T cell exhaustion and prostate cancer progression. Hum Cell. 2025;38(5):131. 40681951 10.1007/s13577-025-01257-0 PMC12274262

[94] Li S, Yi M, Dong B, Jiao Y, Luo S, Wu K. The roles of exosomes in cancer drug resistance and its therapeutic application. Clin Transl Med. 2020;10(8):e257. 33377643 10.1002/ctm2.257 PMC7752167

[95] Raghavan A, Rao P, Neuzil J, Pountney DL, Nath S. Oxidative stress and Rho GTPases in the biogenesis of tunnelling nanotube 34921322 10.1007/s00018-021-04040-0 PMC8683290

[96] Peng Y, Zhao M, Hu Y, Guo H, Zhang Y, Huang Y, et al. Blockade of exosome generation by GW4869 inhibits the education of M2 macrophages in prostate cancer. BMC Immunol. 2022;23(1):37. 35941539 10.1186/s12865-022-00514-3 PMC9361607

[97] Palmulli R, Machesky LM. Is macropinocytosis more than just a passive gulp? Curr Opin Cell Biol. 2025;94:102513. 40220735 10.1016/j.ceb.2025.102513

[98] Shende S, Rathored J, Budhbaware T. Role of metabolic transformation in cancer immunotherapy resistance: molecular mechanisms and therapeutic implications. Discov Oncol. 2025;16(1):453. 40175681 10.1007/s12672-025-02238-3 PMC11965038

[99] You R, Wang B, Chen P, Zheng X, Hou D, Wang X, et al. Metformin sensitizes AML cells to chemotherapy through blocking mitochondrial transfer from stromal cells to AML cells. Cancer Lett. 2022;532:215582. 35122876 10.1016/j.canlet.2022.215582

[100] Liu J, Li X, Li Y, Gong Q, Luo K. Metformin-based nanomedicines for reprogramming tumor immune microenvironment. Theranostics. 2025;15(3):993–1016. 39776799 10.7150/thno.104872 PMC11700864

[101] Velarde F, Ezquerra S, Delbruyere X, Caicedo A, Hidalgo Y, Khoury M. Mesenchymal stem cell-mediated transfer of mitochondria: mechanisms and functional impact. Cell Mol Life Sci. 2022;79(3):177. 35247083 10.1007/s00018-022-04207-3 PMC11073024

[102] Lyu X, Yu Y, Jiang Y, Li Z, Qiao Q. The role of mitochondria transfer in cancer biological behavior, the immune system and therapeutic resistance. J Pharm Anal. 2025;15(3):101141. 40115812 10.1016/j.jpha.2024.101141 PMC11925581

[103] Zhang B, Chang JY, Lee MH, Ju SH, Yi HS, Shong M. Mitochondrial stress and mitokines: therapeutic perspectives for the treatment of metabolic diseases. Diabetes Metab J. 2024;48(1):1–18. 38173375 10.4093/dmj.2023.0115 PMC10850273

[104] Han X, Wang X. Opportunities and challenges in tunneling nanotubes research: how far from clinical application? Int J Mol Sci. 2021;22(5):2306. 33669068 10.3390/ijms22052306 PMC7956326

[105] Islam MN, Das SR, Emin MT, Wei M, Sun L, Westphalen K, et al. Mitochondrial transfer from bone-marrow-derived stromal cells to pulmonary alveoli protects against acute lung injury. Nat Med. 2012;18(5):759–65. 22504485 10.1038/nm.2736 PMC3727429

[106] Hayakawa K, Esposito E, Wang X, Terasaki Y, Liu Y, Xing C, et al. Transfer of mitochondria from astrocytes to neurons after stroke. Nature. 2016;535(7613):551–5. 27466127 10.1038/nature18928 PMC4968589

[107] Watkins SC, Salter RD. Functional connectivity between immune cells mediated by tunneling nanotubes. Immunity. 2005;23(3):309–18. 16169503 10.1016/j.immuni.2005.08.009

[108] Ariazi J, Benowitz A, De Biasi V, Den Boer ML, Cherqui S, Cui H, et al. Tunneling nanotubes and gap junctions–their role in long-range intercellular communication during development, health, and disease conditions. Front Mol Neurosci. 2017;10:333. 29089870 10.3389/fnmol.2017.00333 PMC5651011

[109] Cabrera M, Echeverria E, Lenicov FR, Cardama G, Gonzalez N, Davio C, et al. Pharmacological Rac1 inhibitors with selective apoptotic activity in human acute leukemic cell lines. Oncotarget. 2017;8(58):98509–23. 29228706 10.18632/oncotarget.21533 PMC5716746

[110] Sun J, Gaidosh G, Xu Y, Mookhtiar A, Man N, Cingaram PR, et al. RAC1 plays an essential role in estrogen receptor alpha function in breast cancer cells. Oncogene. 2021;40(40):5950–62. 34373577 10.1038/s41388-021-01985-1 PMC8497275

[111] Liang J, Oyang L, Rao S, Han Y, Luo X, Yi P, et al. Rac1, a potential target for tumor therapy. Front Oncol. 2021;11:674426. 34079763 10.3389/fonc.2021.674426 PMC8165220

[112] Carrizzo A, Vecchione C, Damato A, di Nonno F, Ambrosio M, Pompeo F, et al. Rac1 pharmacological inhibition rescues human endothelial dysfunction. J Am Heart Assoc. 2017. 10.1161/JAHA.116.004746. 28246076 10.1161/JAHA.116.004746 PMC5524008

[113] Grossmann D, Berenguer-Escuder C, Chemla A, Arena G, Kruger R. The emerging role of RHOT1/Miro1 in the pathogenesis of Parkinson’s disease. Front Neurol. 2020;11:587. 33041957 10.3389/fneur.2020.00587 PMC7523470

[114] Zeng L, Yang J, Zhang C, Zhu J, Zhong S, Liu X, et al. Miro1: a potential target for treating neurological disorders. Neuroscience. 2025;577:228–39. 40403957 10.1016/j.neuroscience.2025.05.019

[115] Lu G, Lai Y, Wang T, Lin W, Lu J, Ma Y, et al. Mitochondrial fission regulator 2 (MTFR2) promotes growth, migration, invasion and tumour progression in breast cancer cells. Aging (Albany NY). 2019;11(22):10203–19. 31740625 10.18632/aging.102442 PMC6914410

[116] Zhu H, Wang G, Zhu H, Xu A. MTFR2, a potential biomarker for prognosis and immune infiltrates, promotes progression of gastric cancer based on bioinformatics analysis and experiments. J Cancer. 2021;12(12):3611–25. 33995638 10.7150/jca.58158 PMC8120185

[117] Filichia E, Hoffer B, Qi X, Luo Y. Inhibition of Drp1 mitochondrial translocation provides neural protection in dopaminergic system in a Parkinson’s disease model induced by MPTP. Sci Rep. 2016;6:32656. 27619562 10.1038/srep32656 PMC5020318

[118] Yang J, Chen P, Cao Y, Liu S, Wang W, Li L, et al. Chemical inhibition of mitochondrial fission via targeting the DRP1-receptor interaction. Cell Chem Biol. 2023;30(3):278-94.e11. 36827981 10.1016/j.chembiol.2023.02.002

[119] Cereghetti GM, Costa V, Scorrano L. Inhibition of Drp1-dependent mitochondrial fragmentation and apoptosis by a polypeptide antagonist of calcineurin. Cell Death Differ. 2010;17(11):1785–94. 20489733 10.1038/cdd.2010.61 PMC3000862

